# Univariate and multivariate plasticity in response to incubation temperature in an Australian lizard

**DOI:** 10.1242/jeb.244352

**Published:** 2022-11-28

**Authors:** Madeleine J. de Jong, Craig R. White, Bob B. M. Wong, David G. Chapple

**Affiliations:** School of Biological Sciences, Monash University, Melbourne, 3800 VIC, Australia

**Keywords:** Developmental environment, *Lampropholis delicata*, Multivariate phenotype, Phenotypic integration, Phenotypic plasticity, Thermal physiology

## Abstract

Environments, particularly developmental environments, can generate a considerable amount of phenotypic variation through phenotypic plasticity. Plasticity in response to incubation temperature is well characterised in egg-laying reptiles. However, traits do not always vary independently of one another, and studies encompassing a broad range of traits spanning multiple categories are relatively rare but crucial to better understand whole-organism responses to environmental change, particularly if covariation among traits may constrain plasticity. In this study, we investigated multivariate plasticity in response to incubation across three temperatures in the delicate skink, *Lampropholis delicata*, and whether this was affected by covariation among traits. At approximately 1 month of age, a suite of growth, locomotor performance, thermal physiology and behavioural traits were measured. Plasticity in the multivariate phenotype of delicate skinks was distinct for different incubation temperatures. Cool temperatures drove shifts in growth, locomotor performance and thermal physiology, while hot temperatures primarily caused changes in locomotor performance and behaviour. These differences are likely due to variation in thermal reaction norms, as there was little evidence that covariation among traits or phenotypic integration influenced plasticity, and there was no effect of incubation temperature on the direction or strength of covariation. While there were broad themes in terms of which trait categories were affected by different incubation treatments, traits appeared to be affected independently by developmental temperature. Comparing reaction norms of a greater range of traits and temperatures will enable better insight into these patterns among trait categories, as well as the impacts of environmental change.

## Teaser

**Summary:** Investigation of multivariate plasticity in response to variation in incubation temperature demonstrates that hot and cool incubation temperatures impact different types of traits, and that there appears to be little evidence that covariation among traits affects phenotypic plasticity.

## INTRODUCTION

The environment experienced by developing embryos can have significant and persistent effects on phenotype and fitness (Dufty et al., 2002; West-Eberhard, 2003). These early life stages are highly sensitive and, as such, developmental plasticity is widely observed across taxa, generates substantial amounts of phenotypic variation and, in some cases, may enable better matching of phenotype to environments (West-Eberhard, 2003; Monaghan, 2008; Moczek et al., 2011; Beaman et al., 2016). Plasticity can vary across environments, traits and populations, and is typically described by reaction norms, which plot phenotype values over environmental variation (Lessells, 2008; Murren et al., 2014). It is also one of the primary ways in which species adjust to rapid environmental change (Hoffmann and Sgrò, 2011; Merila and Hendry, 2014).

Environmental temperatures experienced during development have significant effects on phenotype across taxa; however, this effect is particularly pronounced in ectotherms because of their sensitivity to ambient temperature (Deutsch et al., 2008; Buckley et al., 2012; Eyck et al., 2019). Developmental temperatures affect a range of ectotherm traits, such as plasticity in metabolic rate in mosquitofish, *Gambusia holbrooki* (Seebacher et al., 2014), and sex ratios in populations of green sea turtles, *Chelonia mydas* (Jensen et al., 2018). However, organisms are composed of many traits, and plasticity can therefore also occur in suites of traits that may be associated with one another through genetic, functional or developmental means (Pigliucci, 2003; Royauté et al., 2020). These correlations can occur between life-history traits, including growth, metabolic rate, reproductive output and longevity (White et al., 2022), behaviours, such as boldness and aggression in three-spined sticklebacks, *Gasterosteus aculeaus* (Bell and Sih, 2007), as well as between life-history, behavioural and physiological traits, as demonstrated in domestic dogs, *Canis familiaris*, where lifespan was negatively correlated with boldness and aggression was positively correlated with energy expenditure (Careau et al., 2010). With the exception of a few model organisms, however, developmental plasticity has so far been largely explored in the context of single-trait responses (Schlichting and Pigliucci, 1998; Pigliucci, 2005; Plaistow and Collin, 2014; but see Goodman et al., 2013; Hector et al., 2021). This is surprising because consideration of both univariate and multivariate plasticity, especially across a broad range of trait categories, is crucial if we aspire to better understand whole-organism responses to environmental change (Teplitsky et al., 2014; Warner, 2014; Chirgwin et al., 2015).

Characterising plasticity in the multivariate phenotype may shed light on aspects of development and, specifically, short-term responses to environmental change that may otherwise be difficult to detect if solely taking a univariate approach. Many traits in ectotherms are closely linked with temperature and may therefore be associated with one another. This includes physiological, behavioural and life-history traits, which are hypothesised to covary with one another along a slow–fast life-history continuum in response to variation in environmental conditions in what is known as a ‘pace-of-life syndrome’ (Reale et al., 2010; Goulet et al., 2017b; Dammhahn et al., 2018). Trait covariance such as that in the pace-of-life syndrome can affect how populations are impacted by environmental change (Moczek et al., 2011; White et al., 2022). This can occur through acting as a potential constraint to plasticity (Pigliucci, 2005; Reale et al., 2010) or, if two traits are positively correlated, a directional shift in the phenotypic expression of one trait may result in a concurrent shift in the correlated trait (Agrawal and Stinchcombe, 2009). As plasticity is vital for coping with environmental change (Hoffmann and Sgrò, 2011), how the multivariate phenotype responds to environmental variation, such as by shifting as a suite of correlated traits or with traits responding independently to environmental differences, will likely affect how species cope with climate warming. Oviparous reptiles are excellent models to address such questions, with a large body of research demonstrating effects of developmental temperature on many independent traits, which can also vary in strength depending on trait type, such as in developmental compared with behavioural traits (Warner, 2014; Noble et al., 2018; While et al., 2018). However, the effect of developmental temperature on multivariate plasticity in reptiles remains largely unexplored.

Here, we investigated univariate and multivariate plasticity in a suite of performance, physiological, developmental and behavioural traits using a model lizard species, the delicate skink, *Lampropholis delicata*, in response to variation in developmental temperature. Delicate skinks are small (34–55 mm adult snout–vent length, SVL) lizards native to eastern Australia (Wilson and Swan, 2021). This species exhibits a pace-of-life syndrome along a cool–hot axis, which positions individuals along a thermal continuum where those with high thermal preference run faster, utilise hotter microhabitats more frequently and are more active, exploratory and bold compared with those with cooler thermal preferences (Goulet et al., 2017a,b; Michelangeli et al., 2018). These associations between behaviour and thermal physiology can arise because individuals that select higher body temperatures may have an increased metabolic rate and therefore more energy for engaging in behaviours such as activity and locomotion (Goulet et al., 2017a,b). Alternatively, more active and exploratory individuals may select higher temperatures to support these more energetically expensive behaviours. Some of these traits have demonstrated plasticity in response to incubation temperature (Downes and Shine, 1999; Bilcke et al., 2006), and, if one or more of these are affected by changes in incubation temperature, associated traits within the pace-of-life syndrome may be simultaneously affected. For example, if a cool incubation temperature resulted in a preference for cooler temperatures, we might expect that individuals would run slower, use cooler microhabitats more frequently and be less active than those from a mild or hot incubation temperature. Hence, we would observe a shift in the multivariate phenotype. Changes in thermal preference may, for instance, be driven by a preference for similar environments to those experienced early in development (natal habitat preference induction: Davis and Stamps, 2004) or by effects of incubation treatment on growth rate and, subsequently, metabolic rates (allocation model of energy budgeting: Careau et al., 2008). According to the allocation model, by selecting cooler temperatures, less energy is required for supporting energetic demands, allowing more energy to be diverted to growth (Careau et al., 2008). This has been found in both roaches, *Rutilus rutilus* (van Dijk et al., 2002), and common minnows, *Phoxinus phoxinus* (Killen, 2014), where individuals that were undergoing a high rate of growth (as compensation for an earlier period of slowed development) also preferred cooler temperatures.

In our study, we measured thermal preference, locomotor performance, microhabitat choice and dispersal in delicate skinks incubated across three incubation temperature treatments to determine whether incubation temperature affects the position of individuals along the cool–hot dimension of the pace-of-life syndrome. Growth rate and metabolic rate of delicate skinks were also measured to examine whether possible shifts along the cool–hot axis were associated with changes in growth and metabolism predicted by the allocation model of energy budgeting. Finally, we examined whether there were any changes in phenotypic covariance matrices in delicate skinks, predicting that because the multivariate phenotype would reflect different positions along a thermal continuum, rather than changing associations among traits, covariance among traits would remain stable among incubation temperature treatments.

## MATERIALS AND METHODS

### Animal collection and husbandry

During the early breeding season (12–27 September) of 2018, gravid *Lampropholis delicata* (De Vis 1888) were collected using hand capture and mealworm fishing methods, which do not bias capture towards specific phenotypes (Michelangeli et al., 2016), from Sydney (33°47′S to 33°54′S, 151°03′E to 151°14′E) and transported to the laboratory at Monash University (Clayton, VIC, Australia). Lizards were housed in temperature-controlled rooms (∼22°C) and were kept individually in plastic containers (25×20×18 cm) lined with newspaper and containing a moist soil laying substrate. Water was available *ad libitum*, and the skinks were fed three times weekly with crickets, *Acheta domesticus*, dusted in vitamin powder. Under each housing container was a heat mat which created a 24 h thermal gradient (21–34°C) and allowed lizards to behaviourally thermoregulate. Lights were switched on between 06:00 h and 20:00 h and were supplemented with UV light. The containers were checked twice daily, in the morning and afternoon, to ensure eggs were collected within 14 h of laying. Animal collection and housing, as well as the following experiments, were approved by the relevant governing bodies (Monash University Animal Ethics Committee: 16757; NSW National Parks and Wildlife Service: SL102124).

### Incubation conditions and hatchling husbandry

Once the eggs were collected, they were weighed individually to the nearest 0.0001 g and placed into small plastic containers with moist vermiculite (1:1.2 vermiculite to water ratio by mass: Downes and Shine, 1999; Holleley et al., 2015), which were covered to prevent water loss. Each egg was assigned a unique number unrelated to incubation treatment. We then split each clutch randomly using a random number generator across three incubators (IC24, Labec, Marrickville, NSW, Australia) set to different temperatures: a cool treatment of 22±3°C, a mild treatment of 26±3°C and a hot treatment of 30±3°C. These treatments were designed to mimic present-day natural nest temperatures found within an urban park in Sydney, with temperatures of 20±3, 26±3 and 28±3°C all recorded within a single park (33°53′S, 151°10′E) (Qualls and Shine, 2000; Bilcke et al., 2006). We selected the intermediate temperature of 26±3°C recorded in natural nests as our mild treatment. The hot incubation treatment, at 2°C above the warm natural nests recorded by Bilcke et al. (2006), was selected to represent upper nest temperatures following predicted climate warming in Syndey by 2050 (Whetton et al., 2015). Finally, the cool treatment mirrored the 4°C difference from the mild temperature that was our hot incubation treatment, with a low temperature of 22±3°C. We checked incubators daily and rotated the eggs between shelves twice per week to minimise any potential effects of thermal gradients within the incubators.

Once the eggs hatched, we weighed the hatchlings to the nearest 0.0001 g, measured their SVL using digital callipers (±0.01 mm), and then housed them individually in plastic containers (25×20×18 cm) in identical conditions. Each container was lined with newspaper, contained an egg carton shelter, and was placed on top of a heat mat to allow behavioural thermoregulation within a 24 h thermal gradient (21–34°C). Lighting (supplemented with UV light) was available between 06:00 h and 20:00 h. Hatchlings were fed three times a week with baby crickets, *A. domesticus*, and water was available *ad libitum*. They were maintained individually in these identical thermal conditions for the duration of the experiment.

### Experimental design

Once skinks reached approximately 4–6 weeks of age, they were measured for a suite of morphological, physiological and behavioural traits, based on previous methods (Goulet et al., 2017a,b; Michelangeli et al., 2017, 2018; Young et al., 2022). These were measured a minimum of 48 h apart, and in an order designed to minimise potential carry-over effects on behaviour, with experiments involving groups of animals performed last (Bell, 2013). The order was as follows: selected temperature, microhabitat choice, resting metabolic rate, sprint speed to estimate thermal performance curves, dispersal, and body size to estimate growth rates. Furthermore, we withheld food in the 24 h prior to testing to standardise digestion levels and video recorded trials where possible. BORIS was used for video playback and scoring of phenotype (Friard and Gamba, 2016). To ensure the observer was blind to incubation treatment, videos were assigned randomly generated codes. We recorded the sex of the lizards by everting the hemipenes once they reached maturity (SVL>30 mm), which was approximately 3–4 months following testing. After this, lizards were maintained in the housing conditions described above for use in future studies.

### Selected temperature

To measure selected temperature, we placed lizards into a 40×100 cm arena divided equally into four runways (Goulet et al., 2017a,b; Michelangeli et al., 2018). Two 250 W infrared bulbs placed over one end of the runways and a temperature-controlled plate under the other end created a thermal gradient ranging from 15 to 36°C. Infrared bulbs were used to eliminate the effect of light as a potential confounding factor. Skinks were placed individually into the centre of a runway and allowed to acclimate for 30 min. Temperature was recorded every 1 min using iButton data loggers (DS1995L-F5+, Maxim Integrated Inc.) placed at 2–3 cm intervals along the runway. Video playback was used to manually record the duration of time spent stopped at different locations along the thermal gradient using BORIS (Friard and Gamba, 2016), only recording instances where skinks were stopped for ≥2 min, which were matched with iButton temperatures. We then calculated mean selected body temperature, weighted for the duration of time stopped, and the range of temperatures selected.

### Microhabitat choice

To measure microhabitat use, following the methods of Michelangeli et al. (2018), we created three types of microhabitat surrounded by sand substrate in large round polyethylene terrariums (110×55 cm; Fig. S1). A terracotta saucer and two large rocks were used to construct the rocky microhabitat, a single small log and moss for the log microhabitat, and plastic grass for the vegetated microhabitat. We placed Petri dishes with water near each microhabitat, as well as a 40 W heat lamp above each to provide basking opportunities. Temperatures were recorded during the experiment with iButtons placed in each microhabitat as well as two in open areas of the arena, which revealed the rocky site was the warmest and the vegetated microhabitat the coolest. Lizards were placed into the arenas individually, allowed to acclimate for 1 h, and then video recorded for 4 h. From these recordings, we manually recorded for each focal lizard the duration of time spent in each microhabitat (s) and the number of transitions between microhabitats (hereafter ‘microhabitat transitions’) using BORIS (Friard and Gamba, 2016) over the 4 h period.

### Resting metabolic rate

We measured resting metabolic rate (RMR) as the rate of CO_2_ production (ml CO_2_ h^−1^) of the lizards at three temperatures (15, 25 and 35°C) in a random order. Food was withheld for 2 days prior to testing (Young et al., 2022), and we recorded the mass of individuals to the nearest 0.0001 g prior to measurement. External air was pumped through columns of Drierite and soda lime to scrub air of water vapour and CO_2_, respectively, prior to entering a mass flow controller (Aalborg, Model GFC17, Orangeburg, NY, USA), which regulated flow rate to 60 ml min^−1^ STPD. Air then flowed into glass respirometry chambers, and the concentration of CO_2_ in air leaving the chambers was then analysed (LI-COR, Model LI-840, Lincoln, NE, USA). An empty respirometry chamber was used to measure the background concentration of CO_2_ for approximately 7 min until the CO_2_ trace stabilised. Skinks were then transferred individually into respirometry chambers, which were placed into an incubator set to the test temperature for the duration of measurement. We recorded CO_2_ production for 1 h 20 min, discarding the first 25 min to prevent handling stress causing artifacts in the data. Following measurement, the chamber containing the lizard was replaced with an empty chamber and we repeated the measurement of background CO_2_. Background rates of CO_2_ production were estimated using linear models fitted to the background CO_2_ concentrations measured prior to and following animal measurements. The lowest CO_2_ concentration over a 5 min period was selected for each measurement, corrected for baseline CO_2_ concentration by subtracting baseline values, and then multiplied by the air flow rate (60 ml min^−1^ STPD) to calculate the rate of CO_2_ production.

### Thermal performance curves

To calculate thermal performance curves, we measured the sprint speed of skinks across five temperatures (15, 20, 25, 30 and 35°C) in a random order. Prior to measurement, lizards were warmed or cooled to the test temperature by placing them in a thermal chamber for 30 min and were then raced individually down a 1 m runway heated to the test temperature while being recorded with a high-speed camera (Goulet et al., 2017a,b; Michelangeli et al., 2018). Each lizard was measured three times at each temperature, with a 30 min rest in the thermal chambers between measurements. Where multiple temperatures were tested on the same day, skinks had a minimum 2 h rest period between temperatures (Logan et al., 2018). The runway was divided into 25 cm segments, and we used video playback to record the time point at which lizards crossed each 25 cm marked line. This was used to calculate the speed of each 25 cm segment, and the fastest intervals were then averaged for each temperature. Individual performance curves were generated for each individual following Phillips et al. (2014) and were bounded at critical thermal limits, 4.7 and 40.8°C, based on published data (Greer, 1989). From these curves, we extracted optimal performance temperature (*T*_opt_), maximum sprint performance (*P*_max_, the speed at *T*_opt_) and performance breadth (*P*_breadth_, the range of temperatures where sprint speed is ≥80% its *P*_max_).

### Dispersal

Dispersal was measured both as latency to disperse and as distance dispersed using large round terrariums (110×55 cm) divided into four equal compartments following Michelangeli et al. (2017). Each compartment contained identical artificial environments: a basking site created with a terracotta saucer and 40 W heat lamp, two rocks either side of the basking site, a Petri dish containing water, and a sand substrate (Fig. S2). Each compartment was connected to the next via a small funnel-shaped tunnel, with only the fourth (final) compartment not connected to the first. Dispersal was measured with animals in groups of three to four (Michelangeli et al., 2017). This was necessary as delicate skinks are a social species, commonly living in groups, and this more closely reflected environmental conditions (Chapple et al., 2016; Michelangeli et al., 2017; M.J.d.J., personal observations). As hatching occurred over a 5 month period, we grouped individuals with similar hatching dates to minimise the effects of differences in age or body size on dispersal, and included skinks from varying treatments where differences in incubation duration allowed. Skinks were marked with different coloured, non-toxic paints to identify individuals on video playback. Each group was acclimated for 30 min in the first compartment, after which the tunnel was opened. Video playback was used to manually record latency (s) to first disperse into the second compartment as well as which compartment was the furthest reached by each focal skink over a 4 h period using BORIS (Friard and Gamba, 2016).

### Growth rate

At the end of the experiments, we weighed skinks to the nearest 0.0001 g and measured SVL using digital callipers (±0.01 mm). We then calculated the mass-specific growth rate as (mass_end_−mass_hatch_)/age_end_ and size-specific growth rate as (SVL_end_−SVL_hatch_)/age_end_.

### Statistical methods

All analyses were performed using R (v.4.0.2: http://www.R-project.org/). To examine the effects of incubation temperature on univariate phenotypes, most traits were fitted with mixed effects models using *lmer* and *glmer* (package lme4: Bates et al., 2015). The exceptions were time to first disperse, where we used *coxme* to fit survival curves (package coxme: https://CRAN.R-project.org/package=coxme), *clmm* to run ordinal regression on distance dispersed (package ordinal: https://CRAN.R-project.org/package=ordinal), and *glmmTMB* for zero-inflated gamma distributions for time spent in the different microhabitats (package glmmTMB: Brooks et al., 2017). Prior to analysis, we checked whether variables met assumptions of normality and heterogeneity, and scaled and transformed them where necessary to meet these assumptions. As the microhabitat transitions variable could not be transformed to meet the equality of variances criterion, a model of unequal variance was applied using *lme* (package nlme: https://CRAN.R-project.org/package=nlme). Incubation treatment and mass were included as fixed effects and maternal identity as a random effect (to account for relatedness among siblings) for all models. For the analysis of RMR, measurement temperature and testing order were also included as fixed effects and lizard ID as a random effect to account for repeated measures. For modelling microhabitat preferences, we included microhabitat type as a fixed effect, and for analysis of both dispersal variables, individual aggression was included as a fixed effect (Michelangeli et al., 2017) and group identity as a random effect. Furthermore, for size-specific growth rate, we replaced hatchling mass with hatchling SVL as a fixed effect. As effects of incubation treatment may vary with mass or sex, we examined whether including sex or interactions among fixed effects improved model fit using both Akaike information criteria (AIC) and likelihood ratio tests (LRTs) (Table S1). We used *emmeans* (package emmeans: https://CRAN.R-project.org/package=emmeans) to estimate marginal means of each incubation treatment group from the fitted models and to perform *post hoc* pairwise comparisons between levels of a significant factor.

To examine differences in the multivariate phenotype between incubation treatments, we performed a non-parametric multivariate analysis of variance (NP-MANOVA) using *lm.rrpp* (package RRPP: Collyer and Adams, 2018) following the protocol of Telemeco and Gangloff (2020). Prior to analysis, all traits were *z*-standardised to a mean of zero and variance of one. We removed individuals with missing values and included a covariance matrix to account for relatedness between individuals that was constructed using *kinship* (package kinship2: https://CRAN.R-project.org/package=kinship2). A series of models were fitted that included incubation treatment, the covariates mass and sex, and their possible interactions. We selected the model with the lowest AIC value (Table S2) and generated predicted means and 95% confidence intervals (CI) for each trait to determine the impact of incubation treatment on the multivariate phenotype. Patterns of individual dispersion within groups were then visualised using principal components analysis (PCA). We then compared phenotypic covariance matrices using common PCA. The jump-up approach of the Flury method was used to compare equality, proportionality and shared principal components of the matrices in a pairwise fashion (Phillips and Arnold, 1999; Roff et al., 2012) using custom­-written scripts (Roff et al., 2012).

## RESULTS

### Incubation temperature effects on univariate phenotypes

A total of 73 eggs were incubated, of which 54 hatched successfully, and 44 hatchlings survived to the end of the experiment (hot *n*=17, mild *n*=15, cool *n*=11). Of these surviving lizards, 18 had two surviving siblings, 14 had one surviving sibling and 12 had no surviving siblings. Incubation treatment did not significantly affect hatching success [mean (±95%CI) cool: 0.78 (−0.28, 1.83), mild: 1.79 (0.54, 3.05), hot: 0.91 (−0.01, 1.83); χ^2^=2.04, *P*=0.361) or sex ratio [cool: −0.03 (−1.34, 1.28), mild: −0.42 (−1.52, 0.68), hot: 3.03 (−1.26, 7.32); χ^2^=2.43, *P*=0.297]. There was a positive effect of egg mass [0.30 (0.13, 0.45); χ^2^=15.05, *P*<0.001] and a significant effect of incubation temperature (χ^2^=12.44, *P*=0.002) on hatchling mass, with hot-incubated hatchlings significantly lighter [0.10 (0.09, 0.10)] than mild-incubated [0.11 (0.10, 0.11); *t*=−3.37, *P*=0.005] but not cool-incubated [0.11 (0.10, 0.11), *t*=2.22, *P*=0.084] hatchlings. In contrast, hatchling SVL did not differ among incubation treatments [cool: 16.2 (15.5, 16.8), mild: 16.3 (15.7, 16.9), hot: 16.1 (15.5, 16.7); χ^2^=0.96, *P*=0.620].

**Fig. 1.**
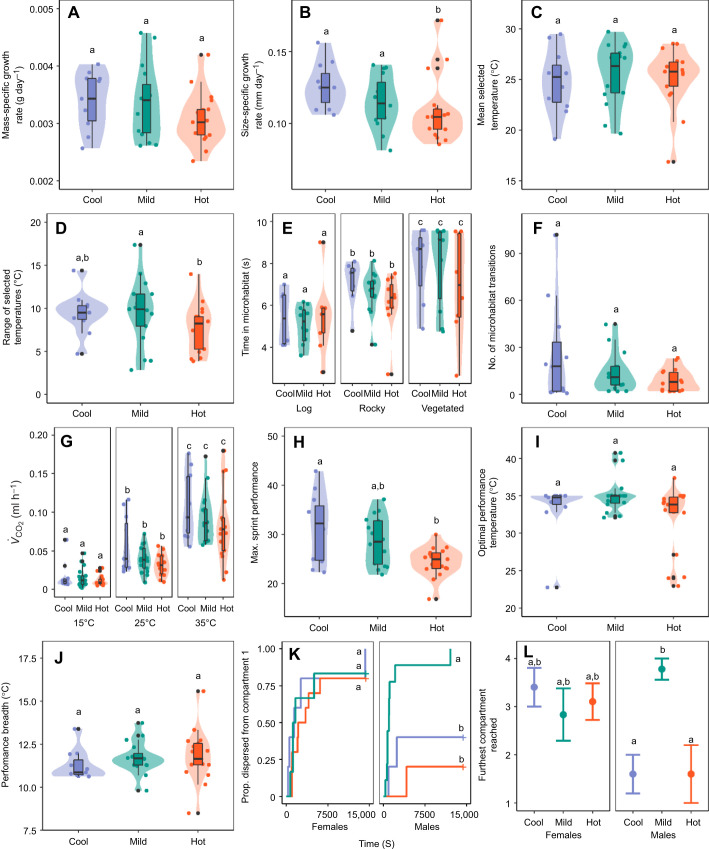
**Effects of incubation temperature on univariate phenotypes in delicate skinks.** Skinks were incubated at cool (blue, *n*=11), mild (green, *n*=15) and hot (red, *n*=17) temperatures. (A–J) Violin and boxplots show medians, interquartile range (IQR) and 1.5× IQR (whiskers), with each point indicating a single measurement for a skink, for mass-specific growth rate (A; incubation treatment: *P*=0.245), size-specific growth rate (B; hatchling snout–vent length, SVL×incubation treatment: *P*=0.024), mean selected temperature (C; incubation treatment: *P*=0.167), range of selected temperatures (D; incubation treatment: *P*=0.027), duration of time within each microhabitat (E; microhabitat×incubation treatment: *P*=0.168), number of microhabitat transitions (F; incubation treatment: *P*=0.611), resting metabolic rate (RMR, as the rate of CO_2_ production, *V̇*_CO_2__; G; incubation treatment: *P*=0.033), maximum sprint performance (H; incubation treatment: *P*=0.002), optimal performance temperature (I; incubation treatment: *P*=0.172) and locomotor performance breadth (J; incubation treatment: *P*=0.145). (K) Survival plot for females and males, showing latency to disperse as the proportion of individuals dispersing for each incubation treatment group over time (sex×incubation treatment: *P*=0.003). (L) The furthest compartment reached for each incubation treatment in the dispersal assay (mean±s.e.m.; sex×incubation treatment: *P*=0.001). Significant differences among groups are indicated by values with different letters (*P*<0.05, adjusted with the Tukey *post hoc* test).

The results of the univariate analyses demonstrated that incubation temperature had variable effects on phenotype (Table S3; Fig. 1). There was a significant effect of treatment on size- but not mass-specific growth rate (Table S3; Fig. 1A,B), the reverse of the results for hatchling morphology. Incubation temperature interacted with hatchling size (SVL) to affect size-specific growth rate. Growth rate of the cool incubation treatment were not affected by hatchling SVL [estimate (±95%CI) 0.004 (−0.01, 0.02)], but the growth rate of mild- and hot-incubated lizards was higher in lizards with smaller hatchling SVL [−0.01 (−0.02, −0.001) and −0.02 (−0.02, −0.01), respectively]. Overall, skinks from the hot incubation treatment [0.10 (0.09, 0.11)] grew significantly slower compared with those from both the cool [0.13 (0.12, 0.14)] and mild [0.12 (0.11, 0.13)] treatments (Tukey's *post hoc* test: *P*=0.002 and *P*=0.017, respectively; Fig. 1B).

Effects of incubation temperature were significant for the range of, but not average, selected temperatures (Table S3; Fig. 1C,D). There was a negative relationship between mass and average selected temperature [−1.02 (−2.03, 0.14); χ^2^=4.08, *P*=0.043]. We also found that skinks from the hot incubation treatment selected a significantly narrower range of temperatures [7.40 (5.84, 8.97)] than those from the mild [10.17 (8.49, 11.86)], but not cool [9.29 (6.94, 11.63)], treatments (Tukey's *post hoc* test: *P*=0.047 and *P*=0.325, respectively), with no significant difference between the cool and mild treatments (*P*=0.810). Neither the number of microhabitat transitions nor the time spent among different microhabitats was significantly affected by incubation temperature (Table S3; Fig. 1E,F). Lizards from all incubation treatments spent more time in the vegetated microhabitat [8.73 (8.24, 9.22)] followed by the rocky [7.04 (6.59, 7.48)] then log [6.04 (5.46, 6.62)] microhabitats. While there was a significant interaction between mass and sex in the main model [0.95 (0.05, 1.86); χ^2^=5.20, *P*=0.023], *post hoc* analysis revealed no significant relationship between mass and microhabitat transitions for either males [0.44 (−0.18, 1.06)] or females [−0.52 (−1.19, 0.15)].

Mass, measurement temperature and incubation temperature all had significant effects on RMR (Table S2; Fig. 1G). Metabolic rate increased with increasing mass [0.25 (0.15, 0.34)] and measurement temperature [15°C: −4.44 (−4.57, −4.30), 25°C: −3.20 (−3.34, −3.07), 35°C: −2.38 (−2.51, −2.24)]. While RMR was highest in cool-incubated skinks [−3.15 (−3.35, −2.95)], it was not significantly different from RMR of hot-incubated [−3.44 (−3.60, −3.29)] or mild-incubated [−3.43 (−3.59, −3.27)] skinks (Tukey's *post hoc* test: *P*=0.061 and *P*=0.091, respectively). We also found a significant effect of incubation temperature on maximum sprint performance (Table S3; Fig. 1H), with cool-incubated skinks being faster sprinters [3.43 (3.32, 3.54)] than hot-incubated [3.20 (3.11, 3.28)], but not mild-incubated [3.33 (3.23, 3.42)], skinks (*post hoc* Tukey test: *P*=0.005 and *P*=0.354, respectively). There was, however, no effect of incubation temperature on optimal performance temperature or performance breadth (Table S3; Fig. 1I,J). Dispersal was significantly impacted by the interaction between incubation treatment and sex (Table S3; Fig. 1K,L). Females maintained high dispersal, in terms of both distance and latency to disperse, regardless of incubation treatment (Fig. 1K,L). In contrast, males incubated at the cool and hot temperatures had a lower dispersal distance [−2.45 (−4.74, −0.15) and −1.77 (−4.06, 0.52), respectively] compared with mild-incubated males [3.58 (1.24, 5.92), Tukey's *post hoc* test: *P*=0.003 and *P*=0.005, respectively]. Cool- and hot-incubated males were also less likely to disperse [−1.57 (−3.02, −0.13) and −2.33 (−4.23, −0.43), respectively] compared with mild-incubated males [1.78 (0.83, 2.63), Tukey's *post hoc* test: *P*=0.003 and *P*=0.003, respectively].

### Effects of incubation temperature on the multivariate phenotype

**Fig. 2.**
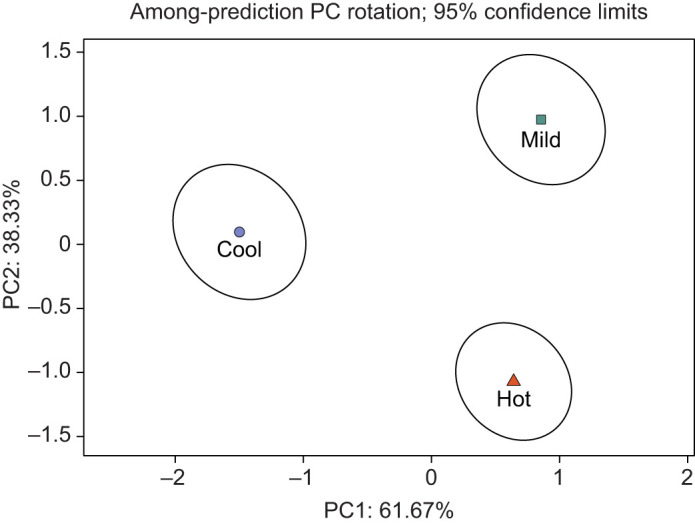
**Principal components analysis (PCA) plot showing the effect of incubation temperature on the multivariate phenotype of delicate skinks.** Plot shows least-squares mean and 95% confidence intervals (Pillai's trace=0.82, *z*=3.52, *P*<0.001) of hot (red triangles, *n*=17), mild (green squares, *n*=15) and cool (blue circles, *n*=11) incubation treatment groups. PCA loadings are provided in Table 3.

We found a significant effect of incubation temperature on the multivariate phenotype of delicate skinks (Pillai's trace=1.27, *z*=3.11, *P*<0.001), with the phenotypes of all three incubation treatments being significantly different from one another (Table 1). Mass, but not sex, was selected as a covariate in the best fitting model, and also had a significant effect on phenotype (Pillai's trace=0.82, *z*=3.52, *P*<0.001). Examination of principal component (PC) plots revealed that PC1 separated the cool incubation group from the mild and hot treatments, and PC2 separated hot-incubated lizards from mild- and cool-incubated lizards (Fig. 2). The variable loadings of predicted values demonstrated that individuals separated along PC1 differed in developmental, performance and physiology traits, as well as in one behavioural trait, dispersal (Table 2). As such, incubation at a cool temperature resulted in individuals characterised by higher RMR, mass- and size-specific growth rates, and maximum sprint performance, but a narrower thermal performance breadth, and reduced dispersal propensity. In contrast, PC2 separated groups along primarily behavioural and performance traits (Table 2), with lizards incubated in the hot treatment described as having a narrower range of selected temperatures, a lower number of microhabitat transitions, decreased dispersal propensity, a lower preference for the warmer rocky microhabitat, a lower maximum sprint performance and a lower optimal performance temperature compared with those incubated in the cool and mild treatments.

**Table 1.**
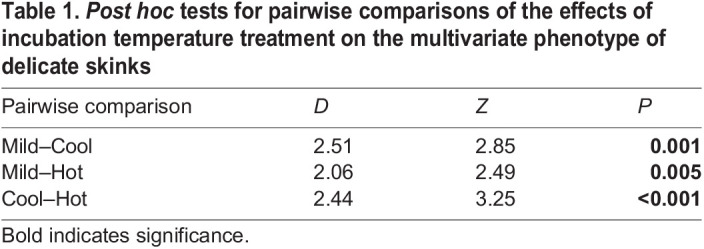
*Post hoc* tests for pairwise comparisons of the effects of incubation temperature treatment on the multivariate phenotype of delicate skinks

**Table 2.**
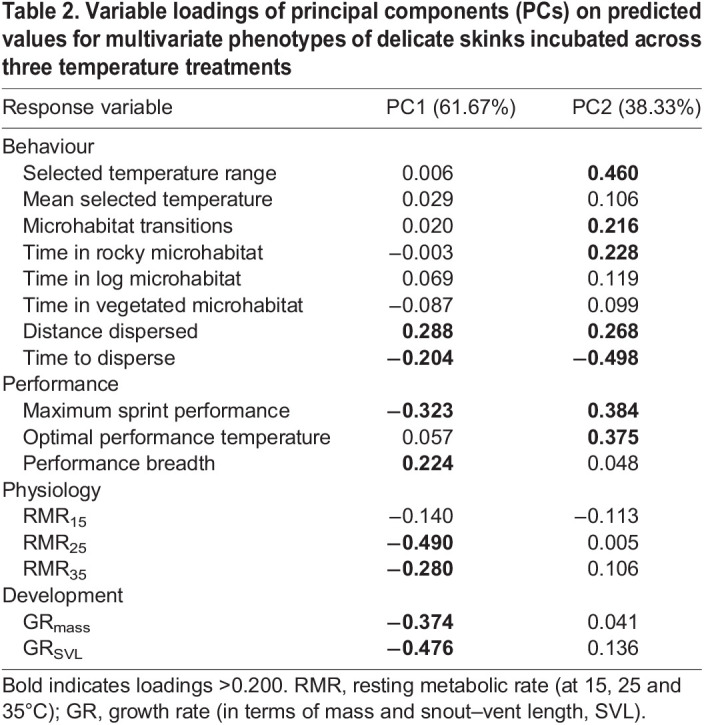
Variable loadings of principal components (PCs) on predicted values for multivariate phenotypes of delicate skinks incubated across three temperature treatments

### Differences in trait covariances among incubation treatments

**Fig. 3.**
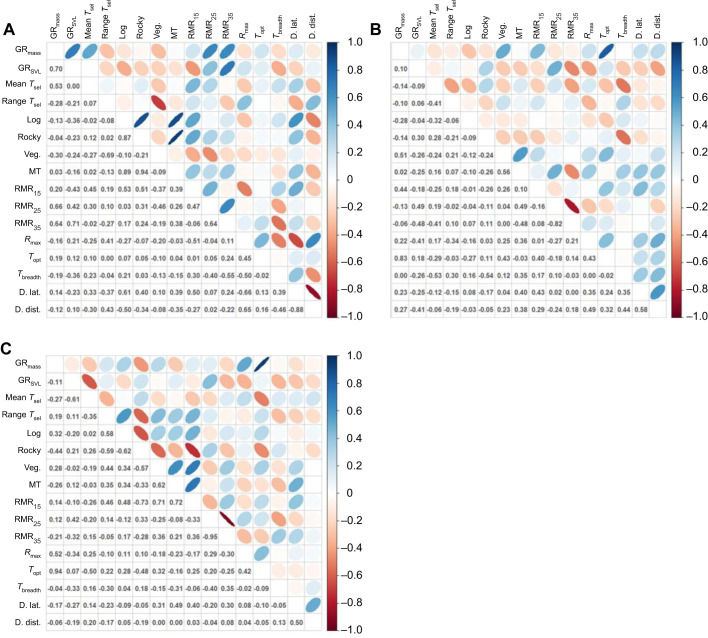
**Plots showing pairwise correlations between behavioural, physiological and growth traits of delicate skinks.** The strength and direction of correlations is shown above the diagonal, and below the diagonal is the correlation estimate for skinks incubated at a cool (A), mild (B) or hot (C) temperature. GR, growth rate (in terms of mass and SVL); *T*_sel_, selected temperature (mean and range); Veg., vegetated microhabitat; MT, microhabitat transitions; RMR, resting metabolic rate (at 15, 25 and 35°C); *R*_max_, maximum sprint performance; *T*_opt_, optimal performance temperature; *T*_breadth_, performance breadth; D. lat., dispersal latency; D. dist., dispersal distance.

Common PCA revealed that the covariance matrices (Fig. 3) did not differ among incubation treatments. We found no differences in matrix equality (*P*>0.599), proportionality (*P*>0.533) or PC (*P*>0.518).

## DISCUSSION

Our results demonstrate that variation among incubation temperatures causes shifts in univariate and multivariate phenotypes. Effects on single traits were variable, but multivariate analysis revealed more clearly defined effects of incubation temperature. The suites of traits affected by treatment differed depending on whether developmental temperature was cool or hot relative to the mild treatment, with impacts of the cool treatment primarily involving development, physiology and performance, whereas a hot incubation temperature had greater effects on behaviour as well as performance. These findings only partially supported our hypothesis that different incubation temperatures would result in phenotypes characterised by a ‘cool’ or ‘hot’ thermal type, as predicted by the cool–hot dimension of the pace-of-life syndrome proposed by Goulet et al. (2017b). Differences in reaction norms are likely the best explanation for differences among treatments in univariate and multivariate plasticity. Overall, our findings suggest that incubation temperature causes plasticity in mean phenotypic expression of behavioural, physiological, performance and developmental traits, with few internal constraints on plasticity.

Differences in incubation temperature affect a broad range of traits and, while they vary in plasticity, most trait categories are affected in some way (Booth, 2006; Mitchell et al., 2018; Noble et al., 2018; While et al., 2018; Refsnider et al., 2019). Here, we also found plasticity in delicate skink phenotypes across multiple categories, with impacts on development, physiology, performance and behaviour in response to variation in incubation temperature. While the results of univariate and multivariate analyses broadly matched, the multivariate analysis of variance captured differences among treatments in traits that did not reveal plasticity when examined independently, such as microhabitat transitions. The former also more clearly revealed unique differences in phenotype that depended on the direction of temperature change. Telemeco and Gangloff (2020) had a similar finding when comparing univariate and multivariate analyses of physiological stress responses in eastern fence lizards, *Sceloporus undulatus*, where different types of stressors impacted different suites of biomarkers, a conclusion which could not be ascertained based on the univariate results alone. In our study, delicate skinks incubated at a cool temperature expressed plasticity in a multivariate phenotype that, for the most part, involved changes in a different set of traits to that of skinks incubated at a hot temperature.

We found that cool-incubated delicate skinks appeared to have a phenotype largely driven by plasticity in growth rate. While this partially supported our hypothesis, as it involved traits such as growth, metabolic rate, locomotor performance and dispersal, there were no effects on thermal preference or microhabitat transitions. Growth rate can increase after exposure to a period of cooler developmental temperatures, which has been demonstrated in green anoles, *Anolis carolinensis* (Goodman, 2008), multi-ocellated racerunners, *Eremias multiocellata* (Ma et al., 2018), as well as a broad range of other ectotherms (Atkinson, 1994; Burraco et al., 2020). Higher growth rates have been linked to higher metabolic rates (Ma et al., 2018; Hazard et al., 2019; Burraco et al., 2020), which is then theorised to affect behaviour and performance through energy availability and allocation (Metcalfe and Monaghan, 2001; Careau et al., 2008; Killen, 2014). As a result, we had expected traits such as microhabitat transitions and selected temperatures to also covary alongside growth and metabolic rates. However, associations between pace-of-life traits appear to be variable (Royaute et al., 2018), and there appear to be inconsistent associations between metabolic rate and behaviour as well as other fitness proxies (Mathot et al., 2019; Arnold et al., 2021). It may be that these correlations vary depending on context, particularly with regards to resource availability and body condition. This may explain why some traits such as thermal preference and activity are part of a pace-of-life syndrome in some studies (Goulet et al., 2017a,b; Michelangeli et al., 2018), but, in others, relationships between traits that are expected to also covary as part of this syndrome are non-existent within the same species (Merritt et al., 2013).

The hot incubation treatment resulted in a multivariate phenotype primarily differentiated from other treatments by changes in behaviour and locomotor performance. This included decreased dispersal propensity, microhabitat transitions, time spent in the warmer rocky microhabitat and range of selected temperatures, as well as decreased maximum sprint performance and optimal performance temperature. Plasticity in these traits, without associated changes in growth and metabolic rates, did not align with our predictions of changes primarily being driven by pace-of-life syndrome traits. Instead, this multivariate behavioural phenotype may indicate a shift away from behaviours that expose them to higher, or more variable, temperatures, as well as improved performance in cooler environments. A negative relationship between thermal ecology traits and thermal habitat is not unusual. In populations of rainforest sunskinks, *Lampropholis similis* and *Lampropholis coggeri*, for example, optimal performance temperature and critical thermal maxima are negatively correlated with long-term highest and maximum recent temperatures, respectively (Llewelyn et al., 2016). Munoz et al. (2016) found a similar negative association between optimal performance temperature and thermal habitat across seven Australian skink species. Behaviour may be facilitating this relationship, ensuring that body temperatures are not considerably mismatched to performance optima, or potentially compensating for a lack of plasticity in physiology at higher developmental temperatures (Forsman, 2015). A match between habitat selection and performance traits was demonstrated in closed-litter rainbow skinks, *Carlia longipes*, where incubation at cool temperatures typical of rocky microhabitats resulted in a performance and morphological phenotype suited to rocky habitats, as well as more frequent selection of that habitat (Goodman et al., 2013). If incubation temperature functions as a reliable indicator of future thermal environments, this behavioural phenotype of hot-incubated delicate skinks may reduce the risk of overheating as temperatures increase with climate change (Kearney et al., 2009; Eyck et al., 2019).

A possible consequence of behaviour buffering organisms to extreme temperatures is that selection pressure on heat tolerance and thermal physiology traits may be reduced, potentially slowing adaptation (Bogert, 1949; Baudier et al., 2015; Buckley et al., 2015). Previous studies have found, however, that incubation temperature effects on delicate skink behaviour depended on the temperature the behaviour was observed at, where behaviour such as activity and feeding performance may be higher when developmental and measurement temperatures match (Bilcke et al., 2006; M.J.d.J., C.R.W., B.B.M.W. and D.G.C., unpublished data). Similarly, sprint speed in delicate skinks increased at hotter temperatures in hot-incubated individuals (Downes and Shine, 1999). While this may represent maladaptive plasticity, recent research has also demonstrated that developmental environments can affect acclimation (reviewed in Beaman et al., 2016), including plasticity in traits such as metabolic rate in response to temperature variation (Seebacher and Grigaltchik, 2014). This may explain the differences in results in studies observing behaviour at a single temperature, such as our study, compared with observations at multiple temperatures. Effects of incubation temperature on acclimation may be equally important, if not more so, as changes in mean phenotype, as the ability to maintain more typical behaviour and performance when faced with consistently high temperatures will play a vital role in long-term population persistence under climate change.

While the suites of traits characterising shifts in the multivariate phenotype in cool- and hot-incubated skinks appeared to reflect different trajectories in response to variation in incubation temperature, this did not seem to be the result of shifts along the cool–hot dimension of the pace-of-life syndrome. Interestingly, this resulted in low dispersal propensity in cool- and hot-incubated males with no associated changes to metabolic rates or locomotor performance. Dispersal is affected by numerous internal and external factors, such as behavioural type, body condition and perception of risk in dispersing to an unknown environment (Clobert et al., 2009; Travis et al., 2013), and it may be that incubation temperature indirectly affects delicate skink dispersal through a factor not measured in our study. The differences between treatments in the suite of delicate skink traits affected by incubation temperature may instead be due to variation in reaction norms, as suggested by Noble et al. (2018), the shapes of which can differ in a trait-dependent way (Murren et al., 2014). For a non-linear, concave reaction norm, which typically describes variation in many phenotypes across thermal environments, differences between traits in reaction norm curvature and shape may mean different phenotypic responses, either in magnitude or in direction, in response to the same temperature change. Comparing reaction norms across a greater span of temperatures would provide valuable insight into these patterns and shed more light on which traits are more likely to be affected by rising temperatures (Noble et al., 2018).

We found that patterns of trait covariation did not differ between incubation treatments, either in covariance matrices or in the strength of relationships among traits. This contrasts with a range of studies demonstrating differences in covariance matrices between environments (Sgro and Hoffmann, 2004; Pigliucci, 2005; Fischer et al., 2016; Matesanz et al., 2021). However, others have found instances where they did not differ, as we also observed here. For example, damselfish, *Pomacentrus moluccensis*, acclimated to different water temperatures has similar correlations among activity, boldness and aggression (Biro et al., 2010). Sniegula et al. (2018) likewise found related covariance matrices among populations of damselflies, *Lestes sponsa*, across latitudes. However, when the damselflies were reared in common garden conditions, which were novel to the individuals, Sniegula et al. (2018) observed differences in matrix shape and direction. Novelty in the degree of predation risk of an environment also altered the correlation matrix structure of Trinidadian guppies, *Poecilia reticulata* (Fischer et al., 2016). An absence of changes in trait covariances may indicate constraints in the relationships among traits (Pigliucci, 2005; Armbruster et al., 2014), but given the low number of correlations among traits and differences in suites of traits affected between treatments, we do not think this is likely. Rather, it is possible that our incubation treatments did not meet the novelty or stress threshold to cause these changes in trait covariances, or that the covariances readily dissociate in response to environmental variation.

### Conclusions

We found plasticity in mean phenotypic expression in response to variation in incubation temperature in delicate skinks, with differences in which suites of traits were affected that depended on the direction of incubation temperature change. In contrast to our predictions, these changes did not involve shifts along a hot–cold thermal type continuum of correlated traits. Instead, plasticity was not related to integration and likely reflected differences in reaction norms among traits. Our results add to the growing body of evidence that correlations or integration among traits do not necessarily constrain plasticity (Fischer et al., 2016; Royaute et al., 2018; Jonas and Cioce, 2019; Matesanz et al., 2021). The ability to express plasticity in traits independently of others may mean a reduction in trade-offs and facilitate adaptation to rapidly changing thermal environments with climate change.

## Supplementary Material

10.1242/jexbio.244352_sup1 Supplementary information
