# Supplementary material for: Univariate and multivariate plasticity in response to incubation temperature in an Australian lizard
Source: J Exp Biol. 2022 Nov 28;225(22):jeb244352. doi: 10.1242/jeb.244352 (PMC10112869; doi:10.1242/jeb.244352)
Supplement: Supplementary information [file jexbio-225-244352-s1.pdf]

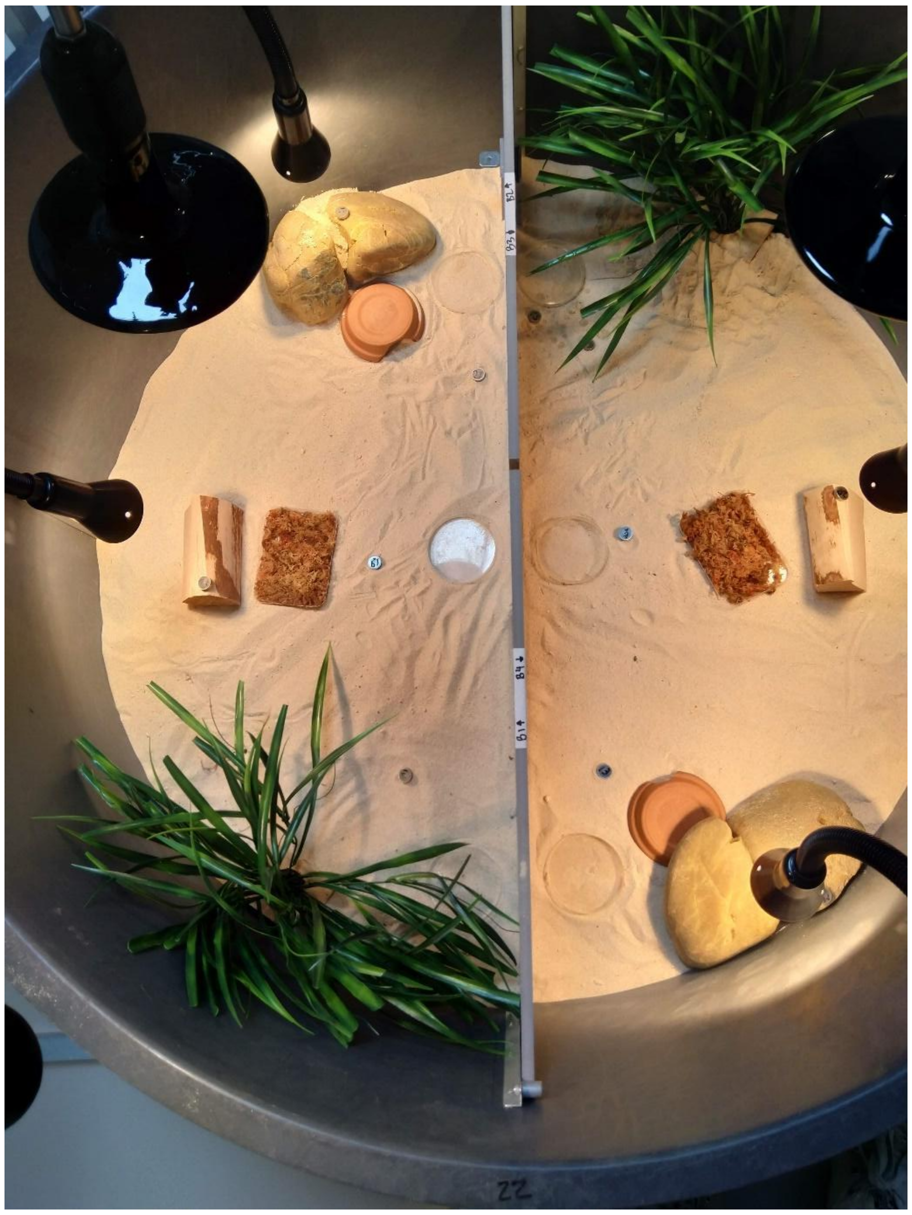

Fig. S1. Experimental set-up for activity and microhabitat use.

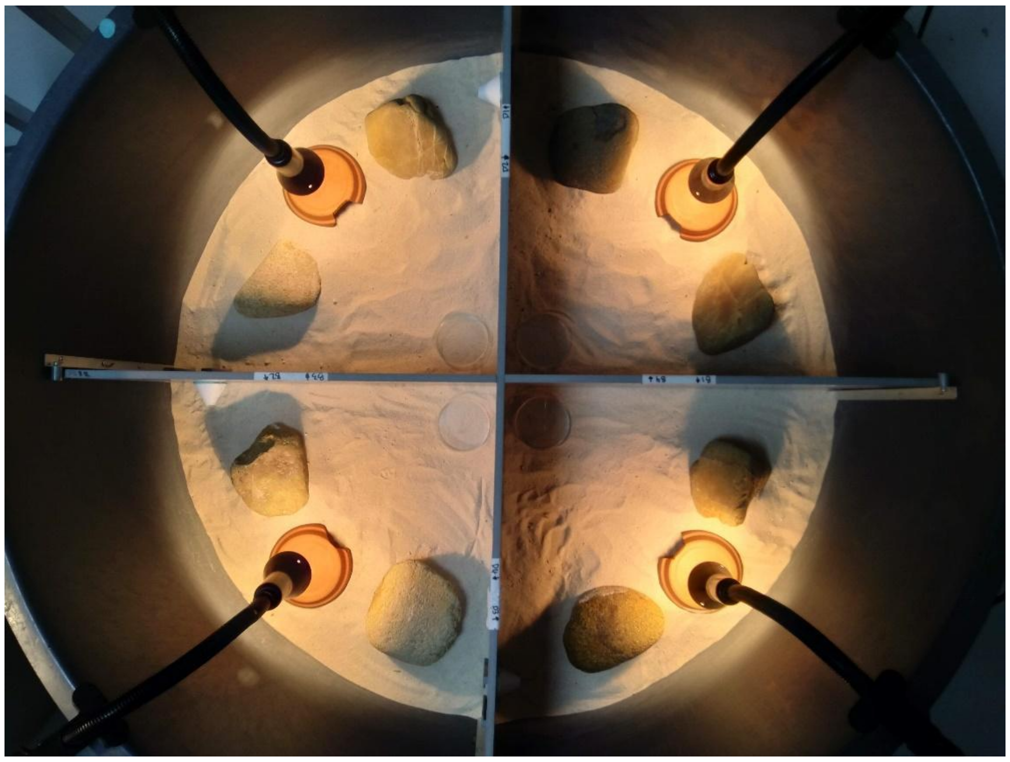

Fig. S2. Experimental set-up for dispersal assays.

Table S1. Corrected Akaike Information Criteria (AICc) model selection table for univariate models to analyse effects of incubation temperature on delicate skink phenotype. Models with delta (Δ) AICc < 2.0 have the greatest support. Models were selected based on both AICc and testing significance of interactions of fixed effects with likelihood ratio tests (LRTs). Bold text indicates the selected models, and italics those models with equal or greater support based on AICc but which were not selected due to results of LRTs. For the variables microhabitat preferences, resting metabolic rate (RMR), and dispersal time and distance, covariates of microhabitat type, testing order and measurement temperature, and individual aggression, respectively, were included as fixed effects in all candidate models.

		Growth rate (mass)	Growth rate (SVL)	Mean T_{sel}	T_{sel} range	Microhabitat preferences	Microhabitat transitions	RMR	R_{max}	T_{opt}	$T_{breadth}$	Dispersal time	Dispersal distance
	Candidate models	ΔAICc	ΔAICc	ΔAICc	ΔAICc	ΔAICc	ΔAICc	ΔAICc	ΔAICc	ΔAICc	ΔAICc	ΔAICc	ΔAICc
1	BS + I	**0.0**	2.0	**0.0**	**0.0**	**0.2**	*0.2*	**0.0**	**0.0**	**0.0**	3.5	*0.0*	5.1
2	BS + I + BS × I	5.6	**0.0**	2.5	5.6	2.4	4.2	4.2	5.3	5.8	8.8	8.3	11.6
3	BS + S + I	5.2	7.7	2.9	2.7	2.2	2.5	2.4	2.9	*0.5*	**0.0**	*1.5*	5.6
4	BS + S + I + BS × I	11.2	6.0	6.0	9.0	3.8	7.0	6.6	8.6	5.6	5.9	16.4	12.2
5	BS + S + I + S × I	10.1	11.5	8.0	4.7	4.4	8.6	6.4	7.6	6.5	4.4	**6.9**	**0.0**
6	BS + S + I + BS × S	11.8	10.6	5.1	6.0	*0.0*	**0.0**	4.4	5.9	3.6	3.1	4.4	9.1
7	BS + S + I + BS × I + S × I	17.2	19.1	13.7	9.9	4.6	13.5	10.7	14.5	12.8	11.8	27.0	6.0
8	BS + S + I + BS × I + BS × S	17.9	12.8	5.2	12.5	4.2	5.2	8.7	11.5	8.9	9.5	18.3	14.7
9	BS + S + I + S × I + BS × S	18.0	16.9	9.4	8.1	3.2	6.9	8.8	10.5	9.9	7.9	8.6	*0.7*
10	BS + S + I + BS × I + BS × S + S × I	26.7	26.7	12.9	12.8	6.8	12.9	13.2	17.4	9.9	15.7	22.1	NA

BS = body size (SVL for growth rate [SVL], mass for all other variables); I = Incubation treatment; S = sex; T_{sel} = selected temperature; R_{max} = maximum sprint performance; T_{opt} = optimal performance temperature; $T_{breadth}$ = Performance breadth.

Table S2. Akaike Information Criteria (AIC) for the candidate models

	Candidate models	AIC	ΔAIC
1	Incubation treatment	1905.79	40.88
2	Incubation treatment + Sex	1923.46	58.55
3	Incubation treatment + Sex + Incubation treatment × Sex	1938.93	74.03
**4**	**Incubation treatment + Mass**	**1864.91**	**0.00**
5	Incubation treatment + Mass + Incubation treatment × Mass	1879.94	15.03
6	Incubation treatment + Sex + Mass	1881.28	16.36
7	Incubation treatment + Sex + Mass + Incubation treatment × Mass	1885.20	20.29
8	Incubation treatment + Sex + Mass + Incubation treatment × Sex	1897.79	32.88

Journal of Experimental Biology • Supplementary information

Table S3. Results and 95% confidence intervals (CI) of linear mixed models analysing the effect of variation in incubation temperature on phenotypic traits in delicate skinks.

Variable	Coefficient	95% CI	χ^2	df	p-value
Growth rate (mass)					
Fixed effects					
Hatchling mass	-1.72×10^{-5}	-2.04×10^{-4}, 1.69×10^{-4}	0.03	1	0.861
Incubation treatment			2.82	2	0.245
Cool inc.	1.92×10^{-5}	-4.16×10^{-4}, 4.54×10^{-4}			
Hot inc.	-3.04×10^{-4}	-7.32×10^{-4}, 1.24×10^{-4}			
Random effects					
Maternal ID	0.00	0.00, 2.56×10^{-4}			
Growth rate (SVL)					
Fixed effects					
Hatchling SVL	-0.01	-0.02, 0.002	**5.30**	**1**	**0.021**
Incubation treatment			**6.48**	**2**	**0.039**
Cool inc.	-0.27	-0.55, -0.001			
Hot inc.	0.04	-0.16, 0.23			
Hatchling SVL × Incubation treatment			**7.47**	**2**	**0.024**
Hatchling SVL × Cool inc.	**0.02**	**0.001, 0.03**			
Hatchling SVL × Hot inc.	-0.003	-0.01, 0.01			
Random effects					
Maternal ID	0.00	0.00, 0.01			
Mean selected temperature					
Fixed effects					
Mass	**-1.02**	**-2.03, 0.14**	**4.08**	**1**	**0.043**
Incubation treatment			3.58	2	0.167
Cool inc.	-2.39	-4.98, 0.12			
Hot inc.	-1.40	-3.38, 0.59			
Random effects					
Maternal ID	0.74	0.00, 2.49			
Selection temperature range					
Fixed effects					

Journal of Experimental Biology • Supplementary information

Mass	-0.98	-2.06, 0.10	3.00	1	0.083
Incubation treatment			**7.20**	**2**	**0.027**
Cool inc.	-0.88	-3.52, 1.79			
Hot inc.	**-2.77**	**-4.82, -0.66**			
Random effects					
Maternal ID	1.52	0.00, 2.88			
Microhabitat preferences					
Fixed effects					
Mass	0.06	-0.25, 0.36	0.13	1	0.719
Microhabitat type			**53.97**	**2**	**< 0.001**
Rocky	**1.64**	**0.76, 2.52**			
Vegetated	**3.59**	**2.63, 4.55**			
Incubation treatment			3.03	2	0.219
Cool inc.	0.73	-0.44, 1.89			
Hot inc.	1.35	-0.33, 3.03			
Microhabitat × Incubation treatment			6.45	4	0.168
Rocky × Cool inc.	-0.22	-1.74, 1.29			
Vegetated × Cool inc.	-0.95	-2.55, 0.65			
Rocky × Hot inc.	-1.71	-3.46, 0.05			
Vegetated × Hot inc.	-1.75	-3.45, -0.05			
Random effects					
Maternal ID	0.32	0.05, 2.05			
ID	0.00	0.00, 0.00			
Microhabitat transitions					
Mass	-0.52	-1.19, 0.15	2.78	1	0.096
Sex (Males)	-0.30	-1.13, 0.54	0.59	1	0.442
Incubation treatment			0.99	2	0.611
Cool inc.	0.16	-0.97, 1.29			
Hot inc.	-0.31	-1.37, 0.75			
Mass × Sex (Males)	**0.95**	**0.05, 1.86**	**5.20**	**1**	**0.023**
Random effects					
Maternal ID	1.64×10^{-4}	-6.06×10^{-206}, 4.46×10^{197}			
Resting metabolic rate					
Order			3.58	2	0.167
Order 2	-0.15	-0.30, 0.004			
Order 3	-0.08	-0.24, 0.07			
Mass	**0.25**	**0.15, 0.34**	**25.69**	**1**	**< 0.001**
Incubation treatment			**6.80**	**2**	**0.033**
Cool inc.	**0.28**	**0.04, 0.52**			
Hot inc.	-0.02	-0.23, 0.19			
Measurement temperature			**719.26**	**2**	**< 0.001**
25	**1.23**	**1.08, 1.38**			
35	**2.06**	**1.91, 2.21**			
Random effects					
Maternal ID	0.05	0.00, 0.23			
ID	0.23	0.06, 0.31			
Maximum sprint performance					
Mass	0.03	-0.03, 0.08	0.94	1	0.333
Incubation treatment			**12.46**	**2**	**0.002**
Cool inc.	0.10	-0.03, 0.24			
Hot inc.	**-0.13**	**-0.25, -0.01**			
Random effects					
Maternal ID	0.00	0.00, 0.14			

Journal of Experimental Biology • Supplementary information

Optimal performance temperature					
Mass	0.53	-0.62, 1.69	0.79	1	0.375
Incubation treatment			3.52	2	0.172
Cool inc.	-1.16	-4.01, 1.70			
Hot inc.	-2.53	-5.12, 0.07			
Random effects					
Maternal ID	0.24	0.00, 2.43			
Performance breadth					
Mass	-0.31	-0.65, 0.05	2.88	1	0.089
Sex			5.89	2	0.052
Males	0.70	0.12, 1.27			
Unknown	0.77	-0.46, 1.87			
Incubation treatment			3.86	2	0.145
Cool inc.	-0.68	-1.40, 0.03			
Hot inc.	-0.09	-0.76, 0.58			
Random effects					
Maternal ID	0.71	0.00, 1.25			
Dispersal time					
Individual aggression	0.41	-0.09, 0.90	2.59	1	0.107
Mass	-0.32	-0.83, 0.19	1.52	1	0.218
Sex (Males)	**1.56**	**0.15, 2.97**	**4.69**	**1**	**0.030**
Incubation treatment			1.66	2	0.437
Cool inc.	0.67	-0.91, 2.25			
Hot inc.	-0.30	-1.76, 0.99			
Sex × Incubation treatment			**11.48**	**2**	**0.003**
Males × Cool inc.	**-3.97**	**-6.66, -1.28**			
Males × Hot inc.	**-3.76**	**-6.52, -1.01**			
Random effects					
Maternal ID	0.68*	NA			
Group ID	0.02*	NA			
Dispersal distance					
Individual aggression	**0.78**	**0.06, 1.51**	**83.04**	**1**	**0.028**
Mass	-0.41	-1.25, 0.44	79.10	1	0.343
Sex (Males)	2.81	-0.04, 5.66	90.09	1	0.093
Incubation treatment			**93.04**	**2**	**0.021**
Cool inc.	1.02	-1.40, 3.44			
Hot inc.	-0.16	-2.20, 1.88			
Sex × Incubation treatment			**89.28**	**2**	**0.001**
Males × Cool inc.	**-7.05**	**-11.64, -2.46**			
Males × Hot inc.	**-5.19**	**-9.14, -1.24**			
Random effects					
Maternal ID	1.14×10^{-5}*	NA			
Group ID	5.47×10^{-7}*	NA			

Bold text indicates significance. *Estimation of 95% CI for random effects not possible for models with > 1 random effect.

Journal of Experimental Biology • Supplementary information
